# *Tribulus Terrestris* for Female Sexual Dysfunction: A Systematic Review

**DOI:** 10.1055/s-0040-1712123

**Published:** 2020-07

**Authors:** Ana Luiza Cabrera Martimbianco, Rafael Leite Pacheco, Fábia Lima Vilarino, Carolina de Oliveira Cruz Latorraca, Maria Regina Torloni, Rachel Riera

**Affiliations:** 1 Department of Medicine, Centro Universitário São Camilo, São Camilo, SP, Brazil; 2 Discipline of Evidence-Based Health, Universidade Federal de São Paulo, São Paulo, SP, Brazil; 3 Centre of Health Technology Assessment, Hospital Sirio-Libanês, São Paulo, SP, Brazil

**Keywords:** tribulus, sexual dysfunction, review, evidence-based medicine, tribulus, disfunção sexual, revisão, medicina baseada em evidências

## Abstract

**Objective** We performed a systematic review to assess the effectiveness and safety of *Tribulus terrestris* to treat female sexual dysfunction (FSD).

**Data sources** We performed unrestricted electronic searches in the MEDLINE, CENTRAL, EMBASE, LILACS, CINAHL, PsycINFO, WHO-ICTR, Clinicaltrials.gov and OpenGrey databases.

**Selection of studies** We included any randomized controlled trials (RCTs) that compared *T. terrestris* versus inactive/active interventions. After the selection process, conducted by two reviewers, 5 RCTs (*n* = 279 participants) were included.

**Data collection** Data extraction was performed by two reviewers with a preestablished data collection formulary.

**Data synthesis** Due to lack of data and clinical heterogeneity, we could not perform meta-analyses. The risk of bias was assessed by the Cochrane Risk of Bias (RoB) tool, and the certainty of evidence was assessed with Grading of Recommendations, Assessment, Development and Evaluations (GRADE).

**Results** After 1 to 3 months of treatment, premenopausal and postmenopausal women randomized to *T. terrestris* had a significant increase in sexual function scores. Three months of treatment with *T. terrestris* showed a significant increase in the serum testosterone levels of premenopausal women. There was no report of serious adverse events, and none of the studies assessed health-related quality of life. The certainty of the evidence was very low, which means that we have very little confidence in the effect estimates, and future studies are likely to change these estimates.

**Conclusion** More RCTs are needed to support or refute the use of *T. terrestris*. The decision to use this intervention should be shared with the patients, and the uncertainties around its effects should be discussed in the clinical decision-making process.

Number of Protocol registration in PROSPERO database: CRD42019121130

## Resumo

**Objetivo** Nós realizamos uma revisão sistemática para avaliar a efetividade e a segurança do *Tribulus terrestris* no tratamento da disfunção sexual feminina (DSF).

**Fontes de dados** Nós realizados uma busca eletrônica irrestrita nas seguintes bases de dados: MEDLINE, CENTRAL, EMBASE, LILACS, CINAHL, PsycINFO, WHO-ICTR, Clinicaltrials.gov, e OpenGrey.

**Seleção dos estudos** Nós incluímos todos os ensaios clínico randomizados (ECR) que comparou *T. terrestris* com controles ativos/inativos. Após o processo de seleção, conduzido por 2 revisores, 5 ECRs (*n* = 279 participantes) foram incluídos.

**Extração de dados** O processo de extração de dados foi realizado por dois revisores, utilizando-se um formulário de extração de dados pré-estabelecido.

**Síntese de dados** Devido à falta de dados disponíveis e à heterogeneidade clínica entre os estudos incluídos, nós não realizamos meta-análises. O risco de viés foi avaliado pela tabela de risco de viés da Cochrane e, a certeza do corpo da evidência foi avaliada pelo *Grading of Recommendations, Assessment, Development and Evaluations* (GRADE).

**Resultados** Após 1 a três 3 meses de tratamento, mulheres na pré e pós-menopausa randomizadas ao *T. terrestris* tiveram um aumento significante nos escores de função sexual. O grupo com 3 meses de tratamento com *T. terrestris* exibiu um aumento significante dos níveis séricos de testosterona em mulheres pré-menopausa. Não houve relato de eventos adversos graves, e nenhum estudo avaliou qualidade de vida das participantes. A certeza da evidência foi considerada muito baixa, o que significa que existe pouca certeza na estimativa dos efeitos e que é provável que futuros estudos mudem estas estimativas.

**Conclusão** Mais ECRs são importantes para apoiar ou refutar o uso do *T. terrestris.* A decisão de usar essa intervenção deve ser compartilhada com pacientes, e as incertezas sobre seus efeitos devem ser discutidas durante o processo de decisão clínica.

## Introduction

Female sexual dysfunction (FSD) is a common condition associated with physical, psychological, and sociocultural factors.^1^ ^2^ ^3^ The International Society for the Study of Women's Sexual Health (ISSWSH) classifies FSD in four categories: hypoactive sexual desire disorder (HSDD), sexual arousal disorders (genital and cognitive), orgasmic disorders, and sexual pain disorders.^4^ In a large epidemiological study conducted over 10 years ago, 12% of 31,581 American women reported a distressing sexual problem, and the percentages were higher among older (45–64 years) participants.^5^ Up to 30 to 50% of women will have FSD during their lifetime, and this rate is probably underestimated due to the social aspects associated with this condition.^3^ ^4^ An estimated 20% of women in all age groups have orgasmic disorders, and 10 to 16% have HSDD, while up to 15% of premenopausal and 30% of postmenopausal women have arousal difficulties.^2^ ^4^ ^6^ ^7^

Standard care for FDS usually involves a multidisciplinary approach, including hormonal therapy, psychotherapy and pharmacological therapy, to address all components of the disorder.^3^ Medicinal plants have been increasingly used by women with FDS, often without a medical prescription.^2^

*Tribulus terrestris* L. (Zygophyllaceae) is a creeping herb, originally from India, which is used as a natural sexual stimulant. *Tribulus* extracts contain protodioscin, a steroidal saponin that can influence hormonal activity and affect the production of endogenous androgen by increasing the release of luteinizing hormone.^2^ ^8^ ^9^ However, the effects of this intervention have not been established. Therefore, the objective of this systematic review was to evaluate the effectiveness and safety of *T. terrestris* for the treatment of FDS (Table 1).

**Table 1:** Main characteristics of the included studies

Study (year)	Participants	Interventions and comparators	Outcomes	Follow-up	Funding
Vale et al (2018)^10^	N = 40 premenopausal women with HSDD Age 18 to 44 years	G1: *T. terrestris* (*N* = 20)* 250 mg orally 3 times/day, 120 days G2: Placebo (*N* = 20)*	Sexual Function (FSFI and SQ-F) Serum testosterone level	Immediately after treatment (4 months)	No financial support
Souza et al (2016)^11^	N = 46 postmenopausal women with HSDD Age 43 to 65 years	G1: *T. terrestris* (*N* = 20)* 250 mg orally 3 times/day, 120 days G2: Placebo (*N* = 16)*	Sexual Function (FSFI) Serum testosterone level	Immediately after treatment (4 months)	A pharmacy provided *T. terrestris* used in the study
Postigo et al (2016)^12^	N = 60 postmenopausal women with HSDD Age: G1 54 ± 5.1 years G2 56 ± 5.8 years	G1: *T. terrestris* (*N* = 30)* 250 mg orally 3 times/day, 90 days G2: Placebo (*N* = 30)*	Sexual Function (SQ-F)	Immediately after treatment (3 months)	The study received funding from a governmental fund and the main investigator had a research fellowship.
Guazzelli et al (2014)^13^	N = 66 postmenopausal women with HSDD Age G1 56 ± 5.8 years G2 53 ± 3.9 years G3 54 ± 5.1 years	G1: *T. terrestris* (*N* = 22)* 250 mg orally 3 times/day, 90 days G2: Tibolone (*N* = 24)* 1.25 mg/oral administration/day, 90 days G3: Placebo (*N* = 20)	Sexual Function (SQ-F)	Immediately after treatment (3 months)	The study received funding from a governmental fund
Akhtari et al (2014)^14^	N = 67 premenopausal women with HSDD Age G1 36 ± 6.2 years G2 36.1 ± 5.8 years	G1: *T. terrestris* (*N* = 30)* 7.5 ml syrup, 2 times / day, 30 days (3.5 g of ethanolic extract per 5 ml of syrup) G2: Placebo (*N* = 30)*	Sexual Function (FSFI) Adverse events	Immediately after treatment (1 month)	The study was supported by Tehran University of Medical Sciences; it is not clear if this was financial support

Abbreviations: FSFI, Female Sexual Function Index; g, grams; HSDD, hypoactive sexual desire disorder; mg: milligrams; ml, milliliters; N, number of participants; SQ-F, Sexual Quotient Female Questionnaire; *T. terrestris, Tribulus terrestris*.

* Number of patients included in the analysis.

## Methods

### Study Design

We registered the protocol of the present study with the International Prospective Register of Systematic Reviews (PROSPERO) (CRD42019121130). The current systematic review of the literature followed the methodological recommendations of the Cochrane Handbook for Systematic Reviews of Interventions^15^ and the reporting recommendations of the Preferred Reporting Items for Systematic Reviews and Meta-Analyses (PRISMA) statement.^16^

### Inclusion Criteria

#### Types of Studies

We included only randomized clinical trials (RCTs).

#### Types of Participants

We included trials that recruited women (aged 16 or over) with a clinical diagnosis of any type of female sexual dysfunction.

#### Types of Interventions

All RCTs that tested *T. terrestris* in any dose, regimen, route of delivery, and for any duration, were eligible for inclusion in the review. The studies had to compare this intervention versus placebo, no intervention, or any active treatment. Trials that administered *T. terrestris* combined with another intervention were eligible if the effects of *T. terrestris* could be isolated.

#### Outcomes

Primary Outcomes:

a) Sexual function assessed by validated tools, such as the Female Sexual Function Index (FSFI)^17^ and Sexual Quotient Female Version (SQ-F).^18^
b) Health-related quality of life assessed by any general or specific validated tool.
c) Serious adverse events defined as the proportion of patients who had at least one life-threating adverse event that resulted in hospitalization, disability or incapacity.
  Secondary outcomes:
d) Serum testosterone levels measured by any laboratory exam.
e) Minor adverse events defined as the proportion of participants presenting at least one minor adverse event.

We considered all time-points reported in the RCTs. We intended to pool (in metanalyses) only similar time points: short term (up to 3 months), middle term (between 3 and 6 months) and long term (more than 6 months).

### Search for Studies

We created a broad and sensitive search strategy, without language, date, or publication status restrictions, to identify all potentially relevant studies.

#### Electronic Search

We ran the search in the following electronic databases to identify studies published from inception to February 11, 2019: MEDLINE (via Pubmed), Cochrane Central Register of Controlled Trials - CENTRAL (via Wiley), EMBASE (via Elsevier), Literatura Latino Americana em Ciências da Saúde e do Caribe - LILACS (via *Biblioteca Virtual em Saúde* [BVS]), Cumulative Index to Nursing and Allied Health Literature – CINAHL (EBSCO host), and PsycINFO (via American Psychological Association). See complete search strategies and all terms used in the searches in Supplementary Table S1.

#### Search for Ongoing Studies

We searched for ongoing studies in the World Health Organization (WHO) International Clinical Trials Registry Platform (apps.who.int/trialsearch) and in ClinicalTrials.gov (www.clinicaltrials.gov).

#### Hand Search and Search for Unpublished Studies

We searched for unpublished studies in Open Gray (http://www.opengrey.eu/). We contacted experts in the field to inquire about any additional ongoing or unpublished studies. We also screened the reference lists of all included studies to identify additional potentially relevant trials.

### Process of Study Selection

We used the Rayyan software^19^ in the two phases of the study selection process. In the first phase, two authors (RLP and COCL) independently screened the titles and abstracts of all records retrieved through the search strategy. In the second phase, the same two authors independently read the full texts of the records coded as ‘potentially relevant’ and included those that fulfilled the aforementioned selection criteria. We created a table with reasons for exclusion of the studies in this phase of the selection process. When needed, a third review author (RR) solved disagreements.

### Data Extraction

We used a data extraction form especially created for this review to collect relevant information from each included trial. Two independent review authors (RLP and ALCM) extracted data; a third author (RR) solved any disagreements.

### Assessment of the Risk of Bias

We used the Cochrane risk of bias tool to assess the methodological quality of the included trials. This tool assesses seven domains of each RCT: random sequence generation, allocation concealment, blinding of participants and personnel, blinding of outcome assessors, incomplete outcome data, selective reporting of outcomes, and other potential sources of bias.^15^ Two authors (RLP and DVP) performed these assessments independently; a third author (RR) solved disagreements.

### Heterogeneity Between Included Studies

We planned to assess the heterogeneity of the intervention effects by visual inspection of the forest plots. We planned to use the chi-squared^2^ test (*p* > 0.1) as indicative of statistical heterogeneity (inconsistency), and the I-squared test to measure the extent of heterogeneity (I^2^ > 50 being indicative of significant heterogeneity).^15^ We also planned to examine the reasons for heterogeneity by conducting additional analyses. This was not possible due to lack of data.

### Measures of Treatment Effect and Analysis

For dichotomous outcomes, we report results using risk ratios (RRs); for continuous outcomes, we used mean differences (MDs). We calculated the 95% confidence intervals (CI) for all reported outcomes. Where possible (availability and homogeneity of data), we planned to pool treatment effects of individual trials into metanalyses using a random effects model and the Review Manager 5.3 software (The Nordic Cochrane Centre/The Cochrane Collaboration, Copenhagen, Denmark).^20^ This was not possible.

### Subgroup and Sensitivity Analyses

We planned to perform subgroup analyses for all primary outcomes comparing pre and postmenopausal women. We also planned to perform two sensitivity analyses for all primary outcomes: random-effects versus fixed-effect metanalyses, and trials with low versus high or unclear risk of selection, detection, performance, and attrition bias. However, due to lack of data, we could not perform these analyses.

### Publication Bias Assessment

We planned to investigate publication bias using funnel plots in metanalyses with more than 10 studies. This was not possible due to lack of data.

### Assessing the Certainty of the Evidence

We used the Grading of Recommendations, Assessment, Development and Evaluations (GRADE) approach 21 to evaluate the certainty of the body of evidence for the comparison between *T. terrestris* versus placebo for the primary outcomes. We assessed the certainty of the evidence in all five GRADE domains (risk of bias, inconsistency, imprecision, indirectness, and publication bias). We report reasons to downgrade or upgrade the evidence. We present a summary of findings table using the software GRADEpro GDT (McMaster University, Hamilton, ON, Canada).^22^

## Results

### Search Results

**Fig. 1:**
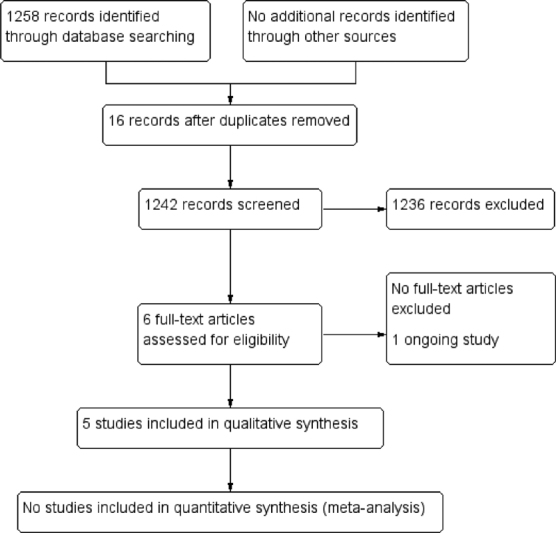
Process of study selection.

The search strategy retrieved 1,258 references. After the exclusion of 16 duplicates, we screened 1,242 unique references, excluded 1,236, and selected 6 for full text reading. One was an ongoing trial (IRCT2016121131340N1) that may contribute data in future updates of this review (Supplementary Table S2). Thus, 5 RCTs were included in the review (Fig. 1). 10 11 12 13 14

### Description of Studies

The 5-parallel design RCTs were published between 2014 and 2017 in Brazil (*N* = 4) and Iran (*N* = 1) and enrolled a total of 279 women with HSDD or loss of libido that caused distress. Three studies^11^ ^12^ ^13^ included only postmenopausal women (*N* = 172; age range 43–65 years), and 2 studies^10^ ^14^ included only premenopausal women (*N*= 107; 18–44 years). Most trials excluded women with any psychiatric condition, smokers, with a history of breast or endometrial cancers, or with diabetes mellitus, cardiovascular or renal disease, and/or using any drugs that could interfere with sexual desire, including hormone therapy. All five RCTs compared *T. terrestris* versus placebo. One study^13^ had three groups: *T. terrestris*, tibolone, and placebo. Four trials administered the drug orally (250 mg 3 times daily for 90–120 days) and 1 gave the participants a syrup containing *T. terrestris* extract (twice daily for 30 days). All five studies assessed sexual function as one of their outcomes; two studies also assessed testosterone levels.^10^ ^11^ Only one study^14^ reported adverse events.

### Risk of Bias of Included Studies

**Fig. 2:**
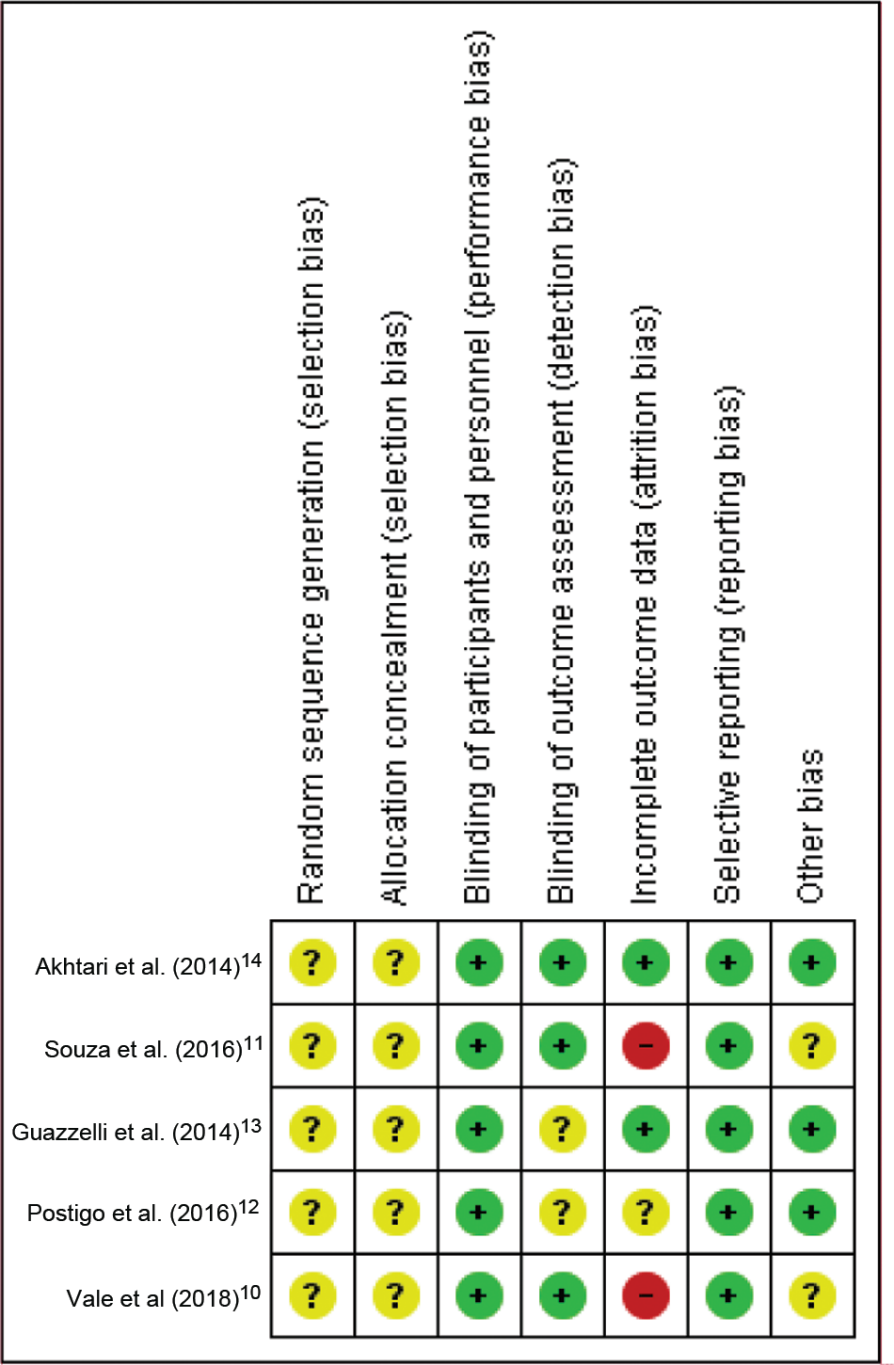
Risk of bias summary.

We classified all trials as having an unclear risk for selection bias (random sequence generation and allocation concealment) because the authors did not provide sufficient information for judgement (Fig. 2). All studies had a low risk of bias for blinding of participants and personnel. Three studies^10^ ^11^ ^14^ had a low risk of bias for blinding of outcome assessors; the other two had an unclear risk of bias for this domain. We classified two studies^10^ ^11^ as having a high risk for attrition bias because of the large number of losses (20% and 37.5%). The five studies reported all the outcomes planned in their registered trial protocols; we, therefore, classified them as having a low risk for reporting bias. Two studies^11^ ^17^ had an unclear risk for other biases because they did not report the baseline characteristics of the study participants.

### Effects of Interventions

Table 2 presents a summary of the results of the five trials. The results of the individual studies could not be combined in metanalyses due to clinical differences in the participants (premenopausal and postmenopausal women) and lack of data (mean and/or standard deviation). We contacted the authors of the studies to obtain additional data but only one replied.^14^

**Table 2:** Summary of the results of the included studies

Outcome		Akhtari et al (2014)^14^		Souza et al (2016)^11^		Guazzelli et al (2014)^13^			Postigo et al (2016)^12^		Vale et al (2018)^10^	
**Primary outcomes** (Final mean (SD))												
		*Tribulus*	Placebo	*Tribulus*	Placebo	*Tribulus*	Placebo	Tibolone	*Tribulus*	Placebo	*Tribulus*	Placebo
**Health-related quality of life**		NA		NA		NA			NA		NA	
**Sexual function** assessed by FSFI (total score)		26.80 (3.03)*	22.41 (2.87)	25.8†	22.93†	NA			NA		25.27†	20.29†
**Sexual function** assessed by: FSFI (sub scores)	Desire	3.09 (0.71)	2.86 (0.79)	3.66†	3.15†						3.24†	2.88†
	Arousal	4.21 (0.67)*	3.17 (0.75)	3.74†	3.04†						3.27†	3.08†
	Lubrication	4.66 (0.87)*	4.18 (0.79)	4.62†	4.39†						3.98†	3.38†
	Orgasm	4.20 (0.72)*	3.59 (0.85)	4.12†	3.83†						3.84†	3.24†
	Satisfaction	4.61 (0.93)*	3.75 (1.12)	4.66†	4.03†						4.36†	3.34†
	Pain	5.07 (1.01)	4.87 (1.42)	5.0†	4.5†						4.58†	4.38†
**Sexual function** assessed by SQ-F (total score)		NA		NA		69†	56†	84†	70.9 (17.6)	56.6 (17.9)	Results were reported based on the presence of sexual dysfunction (%)	
**Serious adverse events**		No cases		NA		NA					NA	
**Secondary outcomes** (Final mean (SD)												
**Serum testosterone levels** assessed by: serum total testosterone level		NA		14.2 (6.9)	11.7 (6.2)	NA					20.5 (9.7)*	13.9 (10.7)
**Minor adverse events**		One patient reported abdominal cramps (not reported in which group)		NA		NA					NA	

Abbreviations: FSFI, Female Sexual Function Index; NA, not assessed; SD, standard deviation; SQ-F, Sexual Quotient Female Version.

* Statistically significant difference.

† Standard deviations were not reported.

#### Sexual Function Assessment

Three studies assessed sexual function using the Female Sexual Function Index (FSFI) (scores range from 2–36, higher values indicate better function), immediately after 1 to 4 10 11 months of treatment in 153 participants (46 post and 107 premenopausal women). Three studies^15^ ^16^ ^17^ used the Sexual Quotient Female Questionnaire (SQ-F) (scores range from 0–100, with higher values indicating better function), to assess sexual function after 3 to 4 months of treatment in 40 premenopausal and 126 postmenopausal women.

**Fig. 3:**
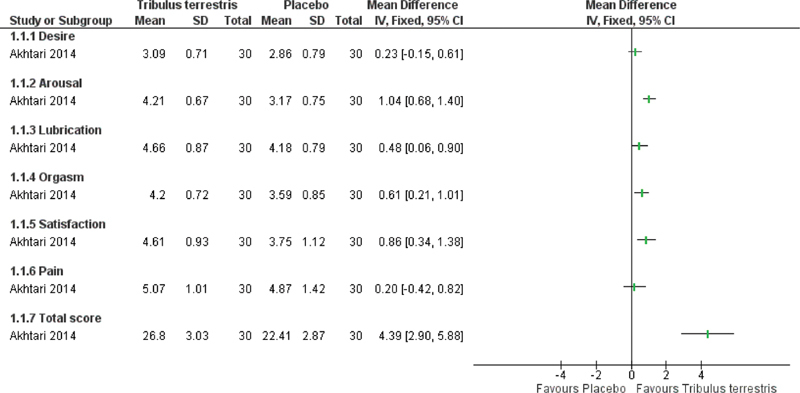
Forest plot of *Tribulus terrestris* versus placebo (only in premenopausal women). Outcome: Sexual function measured by Female Sexual Function Index (FSFI).

One of the three studies that used the FSFI^14^ assessed only premenopausal women and found significantly higher mean total scores in the *T. terrestris* group after 1 month of treatment (mean deviation [MD] 4.39; 95% confidence interval [CI] 2.90–5.88 points; 67 participants; very low certainty evidence). The authors also reported significant increases in arousal, lubrication, orgasm, and satisfaction scores, but not in desire and pain scores (Fig. 3). The other two studies^10^ ^11^ (46 post and 40 premenopausal women) that used the FSFI found non-significant differences in the mean overall scores of the *T. terrestris* and placebo groups after 3 months of treatment (*p* = 0.19 and *p* = 0.44, respectively). These two studies did not provide the standard deviations for these scores (Table 2).

One study^12^ (60 postmenopausal women) found a significant increase in SQ-F scores in the *T. terrestris* group after 3 months of treatment (MD 16.40; 95% CI 7.67–25.13; 60 participants; very low certainty evidence). The other studies^10^ ^13^ (66 postmenopausal and 40 premenopausal women, respectively) reported only the results before and after treatment for each group and did not calculate the differences between them. One of those studies^10^ reported the presence of sexual dysfunction related to each domain of the SQ-F, and there was a significant improvement in all domains after treatment with *T. terrestris* (*p* = 0.001), but not after placebo (*p* = 0.07). The other study^13^ that assessed sexual function using the SQ-F score only reported the final mean scores in each of the three group and did not calculate the differences between them (mean final SF-Q scores: 56 points in the placebo group, 69 in the *T. terrestris* group and 84 in the tibolone group).

#### Adverse Events

Only Akhtari et al (2014)^14^ assessed adverse events. None of the 60 participants had serious adverse events; one participant had abdominal cramps, but the authors did not specify to which group she belonged (60 premenopausal women; very low certainty of evidence).

#### Serum Testosterone Levels

Two studies^10^ ^11^ (96 participants) measured total serum testosterone levels after 3 months of treatment. One study^11^ involved only postmenopausal women and reported non-significant differences between the *T. terrestris* and placebo groups (MD 2.50; 95% CI -1.79–6.79; 46 participants). The other study^10^ involved only premenopausal women and reported a significant increase in testosterone levels in the *T. terrestris* group (MD 6.60; 95% CI 0.27–12.93; 40 participants).

None of the included studies assessed health-related quality of life.

### Certainty of the Evidence Assessment

We assessed the certainty of the evidence for the primary outcomes of the main comparison (*T. terrestris* versus placebo). The certainty of the evidence is very low for sexual dysfunction and adverse events, after 1 and 3 months of treatment. The reasons to downgrade the evidence were the risk of bias of the trials, and imprecision due to small sample sizes. We provide explanations for each judgment in the summary of findings table (Supplementary Table S3).

## Discussion

The present systematic review evaluated the effectiveness and safety of *T. terrestris* in the treatment of women with sexual dysfunction. We identified five RCTs that could not be pooled into metanalyses due to lack of data and differences in study participants. We downgraded the certainty of the evidence to very low due to methodological limitations of the trials and imprecision attributed to small sample sizes. We had concerns about possible selection bias in all trials because the authors provided little information about the methods used for random sequence generation and allocation concealment. Based on the findings of single studies, *T. terrestris*, when compared with placebo, showed an improvement in sexual function scores (FSFI and SQ-F) in premenopausal and postmenopausal women, after 1 to 3 months of treatment. Regarding serum testosterone levels, 3 months of treatment with *T. terrestris* showed a statistically significant increase in premenopausal women, but this effect was not seen in postmenopausal women. Only one study assessed adverse events and reported that one participant had abdominal cramps but did not specify to which group she belonged. Only one study compared *T. terrestris* versus another active intervention (tibolone), but the authors did not provide quantitative data to assess differences between these interventions.

The results of the current review should be interpreted with caution because of the very low certainty of the evidence. This means that we are very unsure about the effect estimates, and future studies are likely to change the magnitude and direction of these estimates. We cannot compare our findings to those of other reviews because, to the best of our knowledge, this is the first systematic review about this intervention.

Our study had several strengths starting with its strict adherence to the methodological recommendations of the Cochrane Handbook and the PRISMA reporting guidelines. We also conducted a broad and sensitive literature search, including gray literature and hand search, to try to identify all potentially relevant studies. The main limitation of the review was the lack of success in obtaining additional information from trial authors. These details would have been important to assess the risk of selection bias of all trials, and additional quantitative data could have allowed us to perform meta-analyses.

The findings of our review should alert clinicians and patients that there is very low certainty evidence regarding the effects (benefits and harms) of *T. terrestris* for the treatment of women with sexual disorders. Current evidence does not support the routine use of *T. terrestris* in clinical practice.

There is a need for additional, well designed, and well conducted RCTs to assess the effects of this intervention for FSD in pre and postmenopausal women. The authors of these trials should adhere to the Consolidated Standards of Reporting Trials (CONSORT) reporting guidelines. This will help to reduce the uncertainty of effect estimates and allow more robust conclusions.

## Conclusion

The present systematic review found very low-certainty evidence, from small single studies, that *T. terrestris* increases sexual function scores (FSFI and SQ-F) in premenopausal and postmenopausal women. However, these results should be interpreted with caution since future studies are likely to change the magnitude and direction of our estimates. More trials are needed to support or refute the use of *T. terrestris* in clinical practice.

## References

[1] Faubion S S Rullo J E Sexual dysfunction in women: a practical approach Am Fam Physician 2015 92 04 281 288 26280233

[2] Mazaro-Costa R Andersen M L Hachul H Tufik S Medicinal plants as alternative treatments for female sexual dysfunction: utopian vision or possible treatment in climacteric women? J Sex Med 2010 7 11 3695 3714. Doi: 10.1111/j.1743-6109.2010.01987.x 20722793 10.1111/j.1743-6109.2010.01987.x

[3] Weinberger J M Houman J Caron A T Anger J Female sexual dysfunction: a systematic review of outcomes across various treatment modalities Sex Med Rev 2019 7 02 223 250. Doi: 10.1016/j.sxmr.2017.12.004 29402732 10.1016/j.sxmr.2017.12.004

[4] Parish S J Meston C M Althof S E Clayton A H Goldstein I Goldstein S W Toward a more evidence-based nosology and nomenclature for female sexual dysfunctions-part III J Sex Med 2019 16 03 452 462. Doi: 10.1016/j.jsxm.2019.01.010 30846116 10.1016/j.jsxm.2019.01.010

[5] Shifren J L Monz B U Russo P A Segreti A Johannes C B Sexual problems and distress in United States women: prevalence and correlates Obstet Gynecol 2008 112 05 970 978. Doi: 10.1097/AOG.0b013e3181898cdb 18978095 10.1097/AOG.0b013e3181898cdb

[6] Aslan E Fynes M Female sexual dysfunction Int Urogynecol J Pelvic Floor Dysfunct 2008 19 02 293 305. Doi: 10.1007/s00192-007-0436-3 17973068 10.1007/s00192-007-0436-3

[7] Frühauf S Gerger H Schmidt H M Munder T Barth J Efficacy of psychological interventions for sexual dysfunction: a systematic review and meta-analysis Arch Sex Behav 2013 42 06 915 933. Doi: 10.1007/s10508-012-0062-0 23559141 10.1007/s10508-012-0062-0

[8] Su L Chen G Feng S G Wang W Li Z F Chen H Steroidal saponins from Tribulus terrestris Steroids 2009 74(4-5):399 403. Doi: 10.1016/j.steroids.2008.12.008 19152803 10.1016/j.steroids.2008.12.008

[9] Basson R Leiblum S Brotto L Derogatis L Fourcroy J Fugl-Meyer K Definitions of women's sexual dysfunction reconsidered: advocating expansion and revision J Psychosom Obstet Gynaecol 2003 24 04 221 229. Doi: 10.3109/01674820309074686 14702882 10.3109/01674820309074686

[10] Vale F BC Zanolla Dias de Souza K Rezende C R Geber S Efficacy of Tribulus Terrestris for the treatment of premenopausal women with hypoactive sexual desire disorder: a randomized double-blinded, placebo-controlled trial Gynecol Endocrinol 2018 34 05 442 445. Doi: 10.1080/09513590.2017.1409711 29172782 10.1080/09513590.2017.1409711

[11] de Souza K Z Vale F BC Geber S Efficacy of Tribulus terrestris for the treatment of hypoactive sexual desire disorder in postmenopausal women: a randomized, double-blinded, placebo-controlled trial Menopause 2016 23 11 1252 1256. Doi: 10.1097/GME.0000000000000766 27760089 10.1097/GME.0000000000000766

[12] Postigo S Lima S MRR Yamada S S dos Reis B F da Silva G M Aoki T Assessment of the effects of tribulus terrestris on sexual function of menopausal women Rev Bras Ginecol Obstet 2016 38 03 140 146. Doi: 10.1055/s-0036-1571472 26902700 10.1055/s-0036-1571472 PMC10309463

[13] Guazzelli R M Lima S MRR Postigo S Martins C PB Yamada S S Estudo dos efeitos do Tribulus terrestris e da tibolona em mulheres com disfunção do desejo sexual após a menopausa Arq Med Hosp Fac Cienc Med Santa Casa São Paulo. 2014 59 01 20 26

[14] Akhtari E Raisi F Keshavarz M Hosseini H Sohrabvand F Bioos S Tribulus terrestris for treatment of sexual dysfunction in women: randomized double-blind placebo - controlled study Daru 2014 22 01 40. Doi: 10.1186/2008-2231-22-40 24773615 10.1186/2008-2231-22-40 PMC4045980

[15] Higgins J Green S, Eds. Cochrane handbook for systematic reviews of interventions [Internet]. Version 5.1.0 London The Cochrane Collaboration 2011 [cited 2019 Aug 10]. Available from: https://handbook-5-1.cochrane.org/

[16] Moher D Liberati A Tetzlaff J Altman D G; PRISMA Group. Preferred reporting items for systematic reviews and meta-analyses: the PRISMA statement BMJ 2009 339 b2535. Doi: 10.1136/bmj.b2535 19622551 10.1136/bmj.b2535 PMC2714657

[17] Rosen R Brown C Heiman J Leiblum S Meston C Shabsigh R The Female Sexual Function Index (FSFI): a multidimensional self-report instrument for the assessment of female sexual function J Sex Marital Ther 2000 26 02 191 208. Doi: 10.1080/009262300278597 10782451 10.1080/009262300278597

[18] Abdo C HN Development and validation of female sexual quotient: a questionnaire to assess female sexual function RBM Rev Bras Med. 2006 63 09 477 482

[19] Ouzzani M Hammady H Fedorowicz Z Elmagarmid A Rayyan-a web and mobile app for systematic reviews Syst Rev 2016 5 01 210. Doi: 10.1186/s13643-016-0384-4 27919275 10.1186/s13643-016-0384-4 PMC5139140

[20] Review Manager (RevMan). Version 5.3 [Computer program] Copenhagen The Nordic Cochrane Centre/The Cochrane Collaboration 2014

[21] Guyatt G H Oxman A D Schünemann H J Tugwell P Knottnerus A GRADE guidelines: a new series of articles in the Journal of Clinical Epidemiology J Clin Epidemiol 2011 64 04 380 382. Doi: 10.1016/j.jclinepi.2010.09.011 21185693 10.1016/j.jclinepi.2010.09.011

[22] McMaster University. GRADEpro GDT: GRADEpro Guideline Development Tool [Computer program] Hamilton Evidence Prime 2015

