# Supplementary material for: Tribulus Terrestris for Female Sexual Dysfunction: A Systematic Review
Source: Rev Bras Ginecol Obstet. 2020 Jul;42(7):427–35. doi: 10.1055/s-0040-1712123 (PMC10316844; doi:10.1055/s-0040-1712123)
Supplement: Supplementary file 1 — Supplementary Material [file 10-1055-s-0040-1712123-s190269.pdf]

Supplementary Table S1 Search strategies for all electronic databases

Medline (via Pubmed)	#1 "Tribulus"[Mesh] OR (Tribulus terrestris) OR (Tribulus terrestri) OR (terrestri, Tribulus) OR (Tribulus muricatus) OR (Tribulus ORientalis) OR (Tribulus lanuginosus) OR (Zygophyllaceae) OR (Goathead) OR (Goatheads) OR (Puncture Vine) OR (Puncture Vines) OR (Vine, Puncture) OR (Vines, Puncture)<br>#2 ((clinical[Title/Abstract] AND trial[Title/Abstract]) OR clinical trials as topic[MeSH Terms] OR clinical trial[Publication Type] OR random*[Title/Abstract] OR random allocation[MeSH Terms] OR therapeutic use[MeSH Subheading])<br>#3 #1 AND #2
Cochrane Central Register of Controlled Trials - CENTRAL (via Wiley)	#1 MeSH descriptor: [Tribulus] explode all trees<br>#2 (Tribulus terrestris) OR (Tribulus terrestri) OR (terrestri, Tribulus) OR (Tribulus muricatus) OR (Tribulus ORientalis) OR (Tribulus lanuginosus) OR (Zygophyllaceae) OR (Goathead) OR (Goatheads) OR (Puncture Vine) OR (Puncture Vines) OR (Vine, Puncture) OR (Vines, Puncture)<br>#3 #1 OR #2<br>#4 #3 In Trials
Embase (via Elsevier)	#1 ''tribulus'/exp OR 'tribulus terrestris'/exp<br>#2 tribulus OR 'tribulus terrestris' OR 'tribulus terrestri' OR 'tribulus muricatus' OR 'tribulus orientalis' OR 'tribulus lanuginosus' OR zygophyllaceae OR goathead OR goatheads OR 'puncture vine' OR 'puncture vines'<br>#3 #1 OR #2<br>#4 [embase]/lim NOT ([embase]/lim AND [medline]/lim)<br>#5 #3 AND #4
Cumulative Index to Nursing and Allied Health Literature – CINAHL (EBSCO host)	#1 Tribulus MH Exact Subject Heading<br>#2Tribulus OR (Tribulus terrestris) OR (Tribulus terrestri) OR (Tribulus muricatus) OR (Tribulus orientalis) OR (Tribulus lanuginosus) OR Zygophyllaceae OR Goathead OR Goatheads OR (Puncture Vine) OR (Puncture Vines)<br>#3 #1 OR #2
Psyc INFO	#1 Any Field: Tribulus OR (Any Field: Tribulus terrestris) OR (Any Field: Tribulus terrestri) OR (Any Field: Tribulus muricatus) OR (Any Field: Tribulus orientalis) OR (Any Field: Tribulus lanuginosus) OR Any Field: Zygophyllaceae OR Any Field: Goathead OR Any Field: Goatheads OR (Any Field: Puncture Vine) OR (Any Field: Puncture Vines)
Literatura Latino Americana em Ciências da Saúde e do Caribe - LILACS (via Biblioteca Virtual em Saúde)	#1 MH":Tribulus terrestris" OR MH:HP4.018.716.673$ OR MH":Tribulus" OR MH: B01.650.940.800.575.912.250.987.849$ OR (Ikshugandha) OR (Abrolhos) OR (Abrolhos-Terrestres) OR (Cruz-de-Malta) OR (Videira da Punctura) OR (Caltrop) OR (Cathead) OR (Yellow Vine) OR (Burra Gokharu) OR (Bindii) OR (Tribulus terrestris) OR (Goatheads) OR (Puncture Vines) OR (Tribulus terrestri) OR (Vine, Puncture) OR (Vines, Puncture) OR (terrestri, Tribulus) OR (Goathead) OR (Puncture Vine) OR (Cadillo) OR (Cuernos de Chivo) OR (Tríbulo) OR (Enredadera Espinuda)
Clinical trials.gov	#1 Tribulus OR Tribulus Terrestris
ICTRP (WHO)	#1 Tribulus OR Tribulus Terrestris

Supplementary Table S2 Characteristic of ongoing studies

Study/ Country	Methods	Participants	Interventions	Outcomes	Status	Last Update
IRCT2016121 131340N1 Iran	RCT Parallel Assignment	Postmenopausal women No limit of age	Experimental (3 groups): Extract of Hydro Alcolol of Teribulus Teristris, Syrup in 0.5, 0.7 and 0.9 - mg/dl, respectively Control: syrups containing mannitol concentration of 0.5 Mg dl Treatment: twice a day for two months	Sexual Function (FSFI Questionnaire) Sexual Satisfaction (Larenson Questionnaire) Time point: 4 and 8 weeks post-treatment	Recruitment Complete (retrospective register)	February 2018

mg/dl, milligram/deciliter; RCT, Randomized clinical trial.

Supplementary Table S3 Summary of findings table

Tribulus terrestris compared with placebo for female sexual dysfunction

Patient or population: Women with sexual dysfunction
Setting: Ambulatory
Intervention: *Tribulus terrestris*
Comparison: Placebo

Outcomes	**Anticipated absolute effects*** (95% CI)		Relative effect (95% CI)	N° of participants (studies)	Certainty of the evidence (GRADE)	Comments
	**Risk with placebo**	**Risk with Tribulus terrestris**				
**Sexual function** Assessed with Female Sexual Function Index (FSFI) Scale from: 2 to 36 Follow up: mean 1 month	The mean sexual function was **2.87** points	The mean sexual function in the intervention group was **4.39** points higher (2.9 higher to 5.88 higher)	–	60 (1 RCT)	⊕◯◯◯ VERY LOW[a,b]	The evidence is very uncertain about the effect of tribulus terrestris on sexual function.
**Sexual function** Assessed with Sexual Quotient Female Questionnaire (SQ-F) Scale from: 0 to 100 Follow up: mean 3 months	The mean sexual function was **54.5** points	The mean sexual function in the intervention group was 16.40 points higher (7.67 higher to 25.13 higher)	–	60 (1 RCT)	⊕◯◯◯ VERY LOW[b,c]	The evidence is very uncertain about the effect of tribulus terrestris on sexual function.
**Serious Adverse events** Follow up: mean 1 month	See comment	See comment	See comment	60 (1 RCT)	⊕◯◯◯ VERY LOW[a,b]	Only one study evaluated adverse events; one participant had abdominal cramp, but authors did not specify to which group she belonged.

*__The risk in the intervention group__ (and its 95% confidence interval) is based on the assumed risk in the comparison group and the **relative effect** of the intervention (and its 95% CI). **CI:** Confidence interval; **MD:** Mean difference

GRADE Working Group grades of evidence
High certainty: We are very confident that the true effect lies close to that of the estimate of the effect
Moderate certainty: We are moderately confident in the effect estimate: The true effect is likely to be close to the estimate of the effect, but there is a possibility that it is substantially different
Low certainty: Our confidence in the effect estimate is limited: The true effect may be substantially different from the estimate of the effect
Very low certainty: We have very little confidence in the effect estimate: The true effect is likely to be substantially different from the estimate of effectQ10

Explanations.
[a]We downgraded one level due to risk of bias (lack of information about random sequence generation and allocation concealment).
[b]We downgraded two levels due to imprecision (small sample size, N = only 60 participants).
[c]We downgraded two levels due to risk of bias (lack of information about random sequence generation, allocation concealment and blinding).
